# Is a Picture Worth a Thousand Words? Using Photo-Elicitation to Study Body Image in Middle-to-Older Age Women With and Without Multiple Sclerosis

**DOI:** 10.1177/10497323211014830

**Published:** 2021-05-24

**Authors:** K. Alysse Bailey, Matthieu Dagenais, Kimberley L. Gammage

**Affiliations:** 1 University of Guelph, Guelph, Ontario, Canada; 2 Brock University, Saint Catharines, Ontario, Canada

**Keywords:** visual methods, disability, positive body image, appearance concerns, aging, qualitative, Canada

## Abstract

In this study, we explored how women with varying relationships to disability and aging used photographs to represent their body image experiences. Seven middle-aged and older adult women with and without multiple sclerosis were asked to provide up to 10 photographs that represented their body image and complete a one-on-one interview. We used reflexive thematic analysis to develop themes and interpret the findings. Overall, the women expressed not only complicated relationships with their bodies, represented through symbolism, scrutiny of body features (e.g., posture, varicose veins, and arthritis) but also deep reflection linked to positive body image and resilience. These findings revealed not only the nuanced experiences women have with aging, disability, and gender but also the commonly experienced ingrained views of body appearance as each participant illustrated a difficult negotiation with the aesthetic dimension of their body image. Finally, we provide important implications of the use of visual methods in body image research.

## Introduction

Qualitative research has made its mark in the body image literature as scholars are increasingly utilizing different methods under various epistemological and ontological orientations. This diversity has allowed the field to expand the populations studied (e.g., Indigenous girls; McHugh et al., 2014), topics explored (e.g., positive body image; Frisén & Holmqvist, 2010), methodologies used (e.g., grounded theory; Wood-Barcalow et al., 2010), and body image programs created (e.g., Body Image Awareness Seminars, https://exerciseandbodyimagelab.com/bias-program; Bailey et al., 2019; Bailey & Gammage, 2020).

Since the call to diversify populations studied in the body image literature (Cash & Pruzinsky, 2002), qualitative research has contributed to understanding the complexity of body image experiences in people outside the commonly explored young female university student experience. For instance, qualitative research with White women aged 50 to 70 years has shown that concerns regarding the effect of aging on body image are prevalent among middle-aged and older adult women and that sociocultural appearance pressures are salient (e.g., Hurd-Clarke & Griffin, 2008), while other qualitative research has indicated that with age, some individuals become less appearance-focused and susceptible to pressures to conform to cultural appearance ideals (e.g., Hogan & Warren, 2012; Tunaley et al., 1999). Using focus groups with an ethnically diverse sample of older adult men and women, Jankowski et al. (2016) found their participants experienced competing and contradictory sociocultural pressures to look age-appropriate while also resisting the effects of aging on appearance.

In addition to the contributions to understanding body image in older adults, qualitative research has also made an undeniable impact on positive body image research. Positive and negative body image were erroneously assumed to operate on the same continuum, but interview research has shown these constructs to be more complex and independent, with distinct characteristics (Frisén & Holmqvist, 2010; Tylka & Wood-Barcalow, 2015; Wood-Barcalow et al., 2010). In interviews with middle-aged and older adult women, Bailey and colleagues (2016) found some participants described experiencing both negative (e.g., dissatisfaction with body weight and shape) and positive (e.g., acceptance and appreciation for the body) body image simultaneously. The use of interviews allowed for a nuanced analysis providing contradictory findings illuminating the complexity of body image experiences in these women.

In addition to expanding the body image field of research across age groups, there is growing literature on the experiences of people with physical disability. These findings seem to be equivocal; a range of body image experiences have been found across various disabilities. For example, in Pfaffenberger and colleagues’ (2011) study, it was found that individuals with a multiple sclerosis diagnosis had significantly poorer body image than those who did not. Similar findings were reported by Taleporos and McCabe (2002), who found that bodily impairment negatively influenced participants’ psychological experiences, feelings, and attitudes toward their bodies, which was noted to be complexly related to negative feedback experienced by the broader social environment (e.g., stigma). In Bailey et al.’s (2015, 2016) studies, people with spinal cord injury described the many ways they experienced positive body image, including learning to accept and appreciate their bodies, but they also mirrored previous findings (e.g., Pfaffenberger et al., 2011; Taleporos & McCabe, 2002) of negative body image, described as related to the stigmatization of disability in society.

It would be particularly fruitful to explore the complex body image experiences in people across age and disability (e.g., multiple sclerosis). To date, scholarship on the aging–disability nexus has not been comprehensively considered (Aubrecht et al., 2020). For instance, in their recently published book, Aubrecht and colleagues provide the important distinction between aging *into* disability (e.g., developing disability through the aging process), and aging *with* disability (e.g., having multiple sclerosis as you age) to help elucidate the different relationships people may have with both aging and disability without collapsing difference. This nexus emerges out of the assumption that creative and generative possibilities develop when aging is considered within disability studies and politics. The connections people have in regard to aging with disability or aging into disability is further complicated by other intersecting identities (e.g., gender) and positionalities with the sociocultural world. These unique intersections, along with powerful political forces, might be more fulsomely captured using a combination of qualitative methods such as photographs and interviews. Interestingly, although the increase in qualitative research across various samples has helped advance the body image field, photo methods have been seldom used (Nash, 2014).

Photo-elicitation, first named by researcher and photographer John Collier (1957), is a research method where photographs are inserted into the research interview. This qualitative method is similar to the popular photovoice methodology developed by Wang and Burris (1997). Photovoice emerged as a participatory action research approach, created to help people identify, represent, and enhance community needs through specific photographic techniques. Photovoice has three main goals: (a) to enable people to record and reflect community needs, (b) promote critical dialogue and disseminate knowledge about important issues through discussion of photographs, and (c) to ultimately reach policymakers to address these needs. This powerful methodology has been taken up to help understand and address the needs of people with intellectual disability (e.g., Watchman et al., 2020) and Crouzon syndrome (e.g., Wheeler & Early, 2018). In Payne et al.’s (2016) photovoice study with young disabled women, participant-produced photographs were useful for demonstrating the daily interactions people with disability encounter. Participants in this study were able to voice the change they wanted, which was for people to see them as young women and not just as disabled.

One key difference with photo-elicitation (as opposed to photovoice) is that photographs can be either interviewee-produced or researcher-produced. The researcher-produced approach is said to be an effective deductive method for theory-driven research (Clark-Ibáñez, 2004). For instance, Gorely et al. (2003) used eight slides of images of female and male track and field athletes and dancers, and representations of female anorexia as “visual triggers” for girls’ and boys’ narratives. They found that in girls’ and boys’ ways of seeing the body, muscularity was a distinctive corporeal dimension of masculinity, but *not* femininity. The images aided in the participants’ articulations of gendered embodiment and helped researchers link these participants’ expressions with feminist and sociocultural theories.

As an alternative example, Nash (2014) used interviewee-produced photographs to provide an inductive approach to understand pregnant women’s body image. The use of photographs and interviews revealed the multiple and often conflicting meanings of body image and pregnancy. Participants used symbolism (e.g., a photograph of a mushroom) as a way to represent the experience of body changes during pregnancy. The author noted inconsistencies by participants, as some expressed holistic and more accepting body image experiences once in the second trimester of pregnancy; however, they also produced photographs with cropped-out heads, suggesting objectification of the body. Photo-elicitation may offer more complex and enriched interview data than interviews alone, providing improved, critical, and even contradictory understandings of intricate body image experiences.

Curry (1986) underscored that the photo-elicitation interview might “tap hidden emotions that would otherwise be missed” (p. 205) and disclose relevant information that would not be revealed using interviews alone or by the researchers’ simple interpretation of participants’ photographs. For instance, Collier (1957) states,

Pictures elicited longer and more comprehensive interviews but at the same time helped subjects overcome the fatigue and repetition of conventional interviews . . . This was its compelling effect upon the informant, its ability to prod latent memory, to stimulate and release emotional statements about the informant’s life. (p. 858)

Furthermore, through the research process, participants can engage with visual methods (e.g., photo-elicitation) to creatively make sense of themselves and to reflect on the ways they create their identities and understand their bodies, not only verbally (e.g., interviews) but also visually (Gauntlett & Holzwarth, 2006). Thus, photo-elicitation offers a useful tool for investigating the complex ways people think about, make sense of, and construct their social worlds (Rose, 2007) and can further our understanding of body image.

Photo-elicitation and photovoice have also been noted as approaches that foster participant empowerment, as participants have autonomy over photo choices and the interview schedule, promoting participant agency throughout the research process in the creation of rich narrative responses (Fleury et al., 2009; Kantrowitz-Gordon & Vandermause, 2016; Oliffe & Bottorff, 2007; Padgett et al., 2013; Richard & Lahman, 2015). On the contrary, researchers should use visual mediums with caution—whether interviewee- or interviewer-produced, images can further perpetuate disciplined, normalized, and regulated ideals of aesthetic beauty (Bordo, 1993). Overall, because photo-elicitation has demonstrated utility in providing in-depth and multiple understandings of phenomena (e.g., Nash, 2014), it may be a useful method to explore more deeply the complex and contradictory body image experiences of middle-aged and older adult women who may have various relationships of aging *with* and *into* disability.

This study used photo-elicitation to explore body image experiences of middle-aged and older adult women with and without multiple sclerosis. We kept recruitment open to women with multiple sclerosis because we wanted to further understand the intersectional body image experiences of gender, age, and disability. We believe the meanings of gender, disability, and aging, which are often taken for granted, can be exposed, and unraveled when considered together in one study. Photographs might be a useful medium to explore social factors, such as stigma, that have been found to be tethered to the body image experiences of older adults and people with disability (e.g., Hurd-Clarke & Griffin, 2008; Taleporos & McCabe, 2002). Therefore, the objective of this study was to explore how middle-aged and older adult women with varying relationships to disability used photographs to represent their body image experiences. The following research question was explored:

**Research Question 1 (RQ1):** How do women with and without multiple sclerosis represent their body image experiences through the use of photographs?

## Method

### Study Design

We used a qualitative design with in-person, semi-structured interviews to support a safe environment for participants to share sensitive information and topics about themselves (Liamputtong, 2009). Safety was fostered by allowing multiple breaks and offering body image resources when needed. To complement the interview data, we used photo-elicitation (Harper, 2002), where the interviewee-produced photographs representing their body image would be discussed in-person during the one-on-one interview.

### Participants

Seven women from Southern Ontario (age range, 57–69 years) were recruited from a university-affiliated exercise facility which is open to members of the community. This facility was chosen because it has accessible programs for middle-aged and older adults, including people with physical disability. The facility had the following specialized programs: Power Cord-Spinal Cord Injury, Power Cord-Multiple Sclerosis, Heart Strong, and SeniorFit. Five participants were from SeniorFit and two were from Power Cord-Multiple Sclerosis. Two participants from Power Cord and one participant from SeniorFit reported having multiple sclerosis (relapse remitting and secondary progressive). All participants were White, community-dwelling, and active members of the center and thus were regularly physically active. Furthermore, they had the economic means to join the center (they paid a monthly membership fee) as well as the capacity to attend in-person exercise sessions.

### Data Collection

Upon university ethics clearance, recruitment posters were placed around the exercise facility and on the facility’s website. Women interested in the study emailed the research team who provided the letter of invitation outlining study details. Those still interested provided written informed consent and received instructions for taking photographs. At this time, they also completed the demographic form where we asked about age, gender, race, height, weight, and program affiliation at the facility, as a way to describe participants and contextualize the findings. This meeting was about 20 minutes and took place at the facility in a private room or at their home, whichever they preferred. Participants were instructed to collect up to 10 photographs that represented their body image, including negative, neutral, and positive body image. Participants used their own camera device (e.g., cell phone camera, IPad®, or digital camera) or used photographs they already had (e.g., in an album). Participants were instructed to take/collect the photographs within 2 weeks of the initial meeting and then email the research team to schedule the one-on-one interview.

Semi-structured interviews, focusing on participants’ photographs and questions about body image took place in a private room at the university or at their own home, according to their preference. At the interview, participants selected a photograph to start the discussion and then gradually went through each photograph to discuss body image experiences (see Table 1 for interview guide). Interviews were audio recorded for transcription purposes. At the conclusion of the interview, participants provided permission and/or instructions on use of the photographs (because participants are the authors of their images). All photographs presented in this article are included with consent by each participant. Participants understood that confidentiality could not be guaranteed if they provided permission to use photographs of themselves.

**Table 1.** Interview Guide.

Overall, how do you view your body? • How do you see and think about your body? • How do you feel and act toward it? How did you find the experience of taking a photograph that represents your body image? • Did you find this process easy, difficult, etc.? Now let’s go over the photograph you would say represents your body image the most? • Why did you select this photograph? • How does this photograph represent your body image? • How does this photograph represent how you *feel* about your body? • How does this photograph represent how you *see* your body? • How does this photograph represent how you *think* about your body? • How might this photograph represent how you *act* toward your body? Now let’s go over some other photographs that you think represent your body image (go over each photo they share). • How does this photograph represent your body image? • How does this photograph represent how you *feel* about your body? • How does this photograph represent how you *see* your body? • How does this photograph represent how you *think* about your body? • How might this photograph represent how you *act* toward your body? What were some of the advantages of using a photograph to capture your body image? What were some of the disadvantages of using a photograph to capture your body image? Overall, what was your experience with using photography as a means to express your body image? Is there anything else you would like to share in regards to your body image or the use of photography to capture body image experiences? Great, thank you!

### Analysis

Audio recordings were transcribed verbatim for analysis purposes. Reflexive thematic analysis was used for identifying, analyzing, and reporting patterns or themes in the data (Braun & Clarke, 2006, 2019), an ideal analysis tool for body image inquiry (Webb et al., 2015). The researchers immersed themselves in the transcripts, reading them carefully multiple times searching for codes and themes. Then, they generated initial codes by reading and making notes on the transcripts. Themes were generated by organizing a long list of codes and searching for representative quotations. Codes were organized into groups, with co-created names (i.e., themes), and then drafted into a thematic map. The team reviewed and defined the themes and refined the thematic map (see Table 2) with a final round of feedback and discussion. All feedback was incorporated into the writing of the results.

**Table 2.** Thematic Map.

Theme Name	Description
Symbolic representations of body image	• Photos used to symbolize body image experiences • Staging of items to represent complex body image experiences • Multiple interpretations of an image (e.g., vibrant kayaks and fog) to represent beauty pressures and aging
Complex stigmas of disability and appearance	• Photos used to depict complex overlapping stigmas of disability and appearance
Critical appraisal of appearance	• Scrutinizing the body through the use of photographs • Harsh negative evaluations of the body
Navigating difficult body image paradoxes	• Conflicting and competing body image experiences (e.g., media literacy and use of photoshop) • Striving for acceptance of the body while simultaneously buying into ageist beauty trends • Critiquing beauty ideals while also buying into beauty pressures, particularly with aging
Uncovering positive body image and resilience	• Positive body image experiences divulged (e.g., acceptance of the aging process) • Photographs used as an educational medium (e.g., teaching granddaughter about body image) • Participants engaged in a reflexive process increasing awareness about how they treat their bodies

It is important to discuss the quality of this qualitative study (e.g., authenticity, trustworthiness, and credibility; Lincoln & Guba, 1986; Smith & McGannon, 2018). First, data are authentically presented as we pulled from participants’ direct quotations and self-produced photographs throughout the results. Although the interviewer-produced photographs approach has been argued as an effective deductive research tool, Clark-Ibáñez (2004) and Rose (2007) state people’s own selection of images might provide a more authentic representation of their contextual and subjective body experiences.

The total data set used for analysis included seven interviews which ranged from 44 to 110 minutes with a total of 70 accompanying photographs. We deemed this to be sufficient data for our purpose as it provided a rich analysis with which to answer our research question (Braun & Clarke, 2019) and is consistent with similarly conducted studies (e.g., Fleury et al., 2009; Kantrowitz-Gordon & Vandermause, 2016; Watchman et al., 2020; Wheeler & Early, 2018). Analysis was conducted inductively, both independently and collectively through weekly meetings and discussions. The overarching framework that guided the analysis was constructivism (for a description, see Lincoln et al., 2011) where the research team’s application of experience and knowledge about body image co-constructed and strengthened the analysis and writing of results.

## Results

Overall, participants’ use of photographs to demonstrate their body image experiences varied. Some participants used images to articulate the complex experiences of disability, aging, and female pressures of obtaining societal beauty standards. However, photos were sometimes used to scrutinize body parts during the interviews. The following themes reflect the participants’ multifaceted body image experiences at the intersections of gender, aging, and disability: (a) symbolic representations of body image, (b) complex stigmas of disability and appearance, (c) critical appraisal of appearance, (d) navigating difficult body image paradoxes, and (e) uncovering positive body image and resilience.

### Symbolic Representations of Body Image

**Figure 1.**
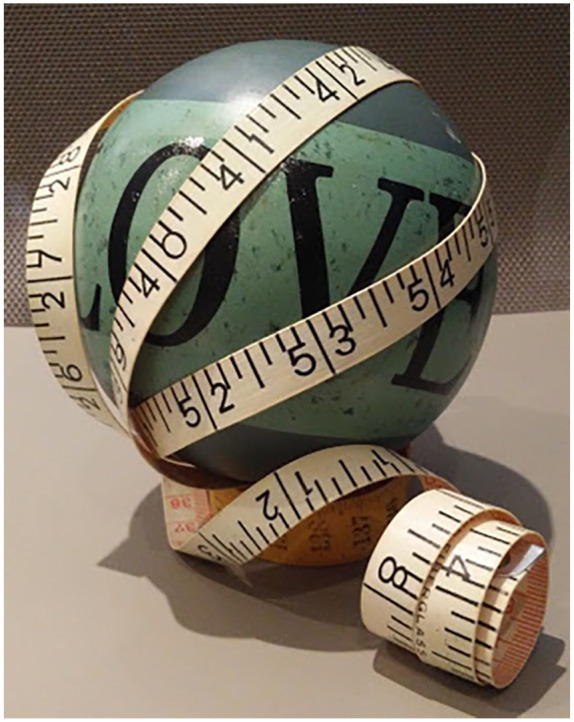
Photograph of a ball and measuring tape.

This theme is represented by women who used photographs to symbolize their body image struggles of aging and Western beauty expectations. For example, one woman used symbolism for the majority of her photographs to represent her body image, taking direct attention away from her body (by not using photographs of herself) but still divulging interesting body image reflections. As a strategy to convey her struggle to love her body, she staged together a ball with tape wrapped around it (see Figure 1). When discussing this approach, she said,

. . . this ball says love, and I chose it because I think, that’s what we are all trying to do, to love ourselves. And the tape is just symbolic of the numbers on it. I think for a lot of people, it’s all about the numbers, people want to be taller, they want to be lighter, they want to be thinner, they want to be smaller, they want to be bigger, and that tape measurement is kind of the tell all for everything. You get wrapped up in it. And that’s why I spun it around the ball. It seems that we can only love ourselves if the numbers are good.

**Figure 2.**
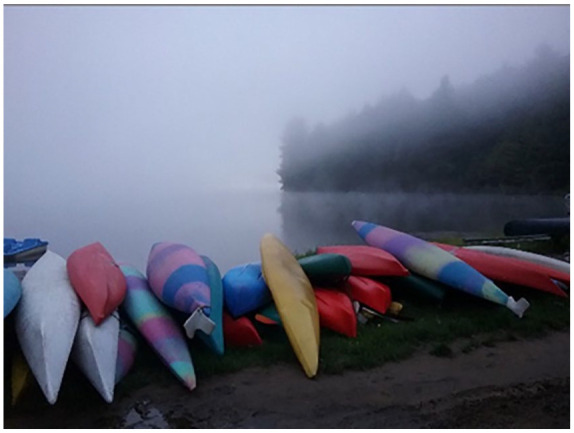
Photograph of kayaks in the fog.

One woman used a photograph as an opportunity to explore a metaphor of aging and feeling invisible (Figure 2 kayaks in the fog). She described this photo as follows:

And one of the photos you’re going to look at is the kayaks in the fog. I’m sure you’d question what that was but I’m finding that if I’m aging, if I stay in my own age group, I still feel vital and alive and all that stuff because comparatively I’m doing alright. But if I’m in a mixed crowd of male, female, and various ages I feel somewhat invisible. And that’s what that was. The vibrant colours of the kayaks and they’re in a bit of fog.

She lamented that when in a mixed group she feels lost in fog, or invisible, due to not quite “measuring up.”

One woman with multiple sclerosis used a photograph she staged of her 20 (and more) beauty products she owns and uses daily. When discussing that photo, she described an exasperated sense of her body as she navigated incessant beauty trends imposed on women, which she reluctantly subscribed to.

### Complex Stigmas of Disability and Appearance

**Figure 3.**
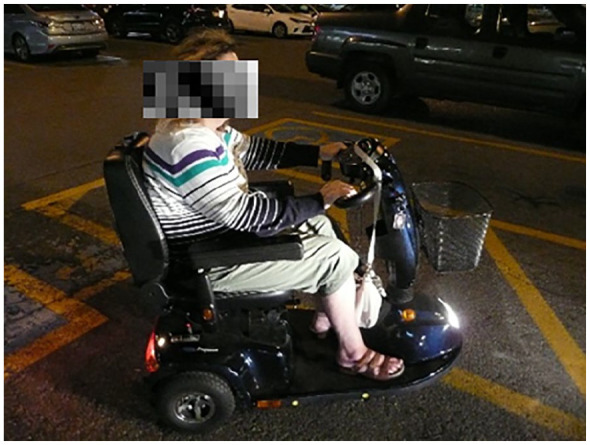
Photograph of the mobility scooter.

In this theme, the women articulated the intricacies of their body image experiences as they pertained to their disability. For instance, one woman with multiple sclerosis (relapse remitting) used a photograph of herself in a mobility scooter to demonstrate the complexities of disability and weight stigma in relation to her body image (see Figure 3). When discussing the significance of this photograph, she stated as follows:

. . . if I want to do something if I want to shop or [go] somewhere further . . . this is my life. It does not make me feel good and a lot of people look [at the scooter] and think “oh it’s because she’s fat.”

She explained feeling stigmatized when shopping or leaving her home and using her mobility scooter. She elaborated,

And more than once I’ll have somebody walking past me saying to me or the person they’re with “oh god I wish I had one of those” and it’s like is it ok for me to say “and I wish I had legs that worked!” You know, to look at them and say can I say this without them being offended . . .?

She also commented “people are always judging you” and “you know because you do see people that are just lazy . . .” When asked if she has felt societal pressures or has overheard people judging her, she stated,

You know they look at you and that’s what I perceive from them if I’m getting into my van in a wheelchair parking spot I have to lift my legs up to get in and you know the people sitting next to me I want them to see that yes there is a reason that I’m in this [scooter] I’m not in it because I like not having to walk. I want people to see that it’s because [of my disability].

Thus, she feared that within public settings people were assuming her use of the scooter is attributed to laziness, a common negative stereotype faced by many people of size (Teachman et al., 2003). During the interview, she explained that she sometimes wished she could hold up a sign stating, “I have a disability” and that is why she uses a scooter, not because she is lazy.

**Figure 4.**
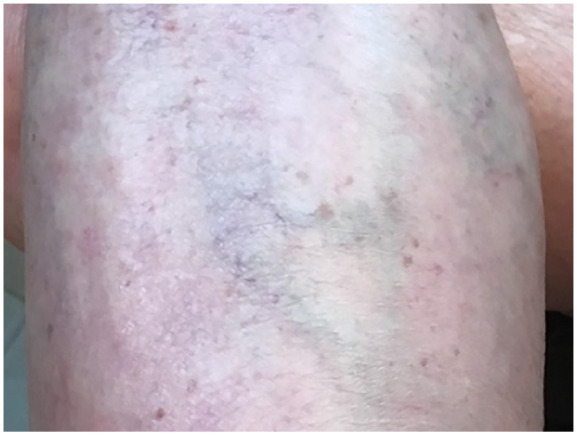
Photograph of varicose veins in the leg.

Another woman who also had multiple sclerosis (secondary progressive) used the photographs to depict aspects of her body image that emphasized her appearance (see Figure 4). She highlighted her legs in photographs and how living with multiple sclerosis has potentially led to changes in her appearance and perceived reactions from others. For instance, she said,

Venous insufficiency. The purple, the lace look. It’s all MS . . . And some people have no filter. So if you’re sitting on the bus at Disney and someone’s looking at you because you’re already in a wheelchair and then they’re looking, you up and down. The way we were brought up, we are so kind to people. If you don’t have anything nice to say, don’t say anything. But when you see them, you know what they’re thinking. That’s something I just hate, but I hate the shape, or I can deal with it a little bit better if the shape was nicer.

She also alluded to the extremes she would take to collapse her varicose veins. She said,

They [the vein doctor] tried to collapse them . . . You sit on the table and she injected each one and it just killed. And then she said, “I really can’t guarantee that you won’t have an MS attack.” I said just do it. Because that was the least of my problems after seeing how they look! But that looks . . . ugh! When she [her caregiver] took that picture, I was like “oh my gosh!”

In this profound statement, she was absorbed in how her varicose veins appeared, even at the cost of serious complications with her disability.

### Critical Appraisal of Appearance

This theme reflects the tendency by women to critically appraise their physical appearance, in this case using their photographs. When describing the photograph she took of her legs, one woman said, “I’m getting those elephant knees haha look at the wrinkles.” She also said,

I wanted to include it because it was something that I look at and go hmm, your legs aren’t what they used to be but on the other hand, it doesn’t bother me that much either. But it’s part of it, it’s still there, it bothers me a little bit but not nearly as much [comparing to what her legs used to look like when she was younger].

Another woman took a similar approach and shared a photograph of her legs. When discussing her displeasure of her legs she said,

And that was recently, I did have shorts on and it just shows why this is not attractive to me, crooked toes with the arthritis, see this toe is crooked and I’ve had to change the shoes I wear too. I can’t wear as pretty shoe as I used to. I can’t wear high heels or narrow shoes. . . . And the knees, this knee is the one that bothers me the most haha because not only is it varicose veins but because of the veins it makes them a bit saggy and so I would just cover this up. I would wear something longer, around the house or gardening I will, I can wear shorts. . .

**Figure 5.**
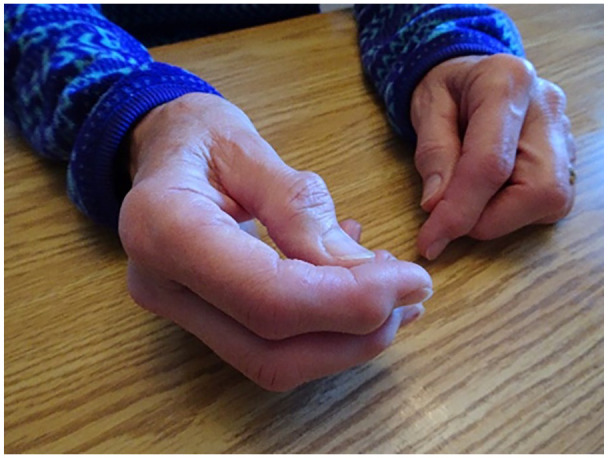
Picture of hands and arthritis.

Throughout much of her interview, she criticized her appearance in the photographs, especially the appearance of her hands (see Figure 5). This echoed the tendency for women to objectify the body and focus on parts rather than the whole. For example, she explained that she is very self-conscious of how her hands look and is preoccupied by it when playing card games with friends. She said,

Because that just shows you how my hands look and I am self-conscious about that. Now, when we play bridge, because I sat and playing bridge again, what I see and what other people see I think they don’t, they just don’t notice but I am very self-conscious of it . . . I go for a manicure now and again but I am always washing my hands so they dry out but I go and the women there, they are nicely manicured and they have these really nice painted nails and I’m thinking, uh oh! . . . Just more worried than I should be.

**Figure 6.**
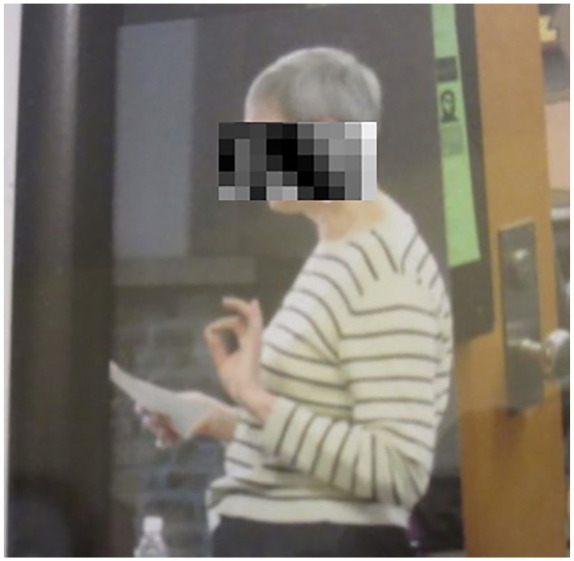
Picture of posture while lecturing.

Another woman with relapse remitting multiple sclerosis discussed her time working at a university giving presentations and lectures to students. When she retired, her colleagues made a collage of her time working there and provided a picture of her lecturing. She was highly critical of this particular photograph, specifically her body posture (see Figure 6). She elaborated as follows:

This is from a collage I have from when I retired that the staff put together. And it’s a, I mean I think it’s a typical picture of me at work with the hands going and I’m very big on hands but it’s this curve that just APPALLED me when I saw it, when I started to see some pictures and I was just appalled. Cause it’s just like, what is that all about?

Similarly, another woman used the photos to scrutinize specific appearance features, particularly her nose. She showed a zoomed in photograph of her nose and used harsh words, such as, “That I hate. Out of everything, the most I hate. Because my sister got a little, cute nose. Hate it. Just hate it, hate the shape, hate the size, hate the point, I hate it.” The emotional disgust she felt toward her nose came across quite strongly during the interview.

### Navigating Difficult Body Image Paradoxes

In this theme, women had conflicting articulations of and relationships with their body image. One woman exemplified this experience by conversing over a photograph that she photoshopped. Early in the interview, she demonstrated media literacy, saying, “. . . cause like you said with media and all that and with advertising, it’s a joke. It’s not what people look like at all.” However, later in the interview, she admitted to often photoshopping images she posted of herself online. When discussing a photograph she took for this study she said,

Now it’s not like the true skin colour or any of the rest of that but if you really wanted to, I could do that at any point in time and I could portray myself as that, no problem. But that’s how little, for me anyway, how little that needs to be changed, in my mind. Considering the whole body area, that’s the only thing I would change. So, but that’s why I did, specifically to show that.

**Figure 7.**
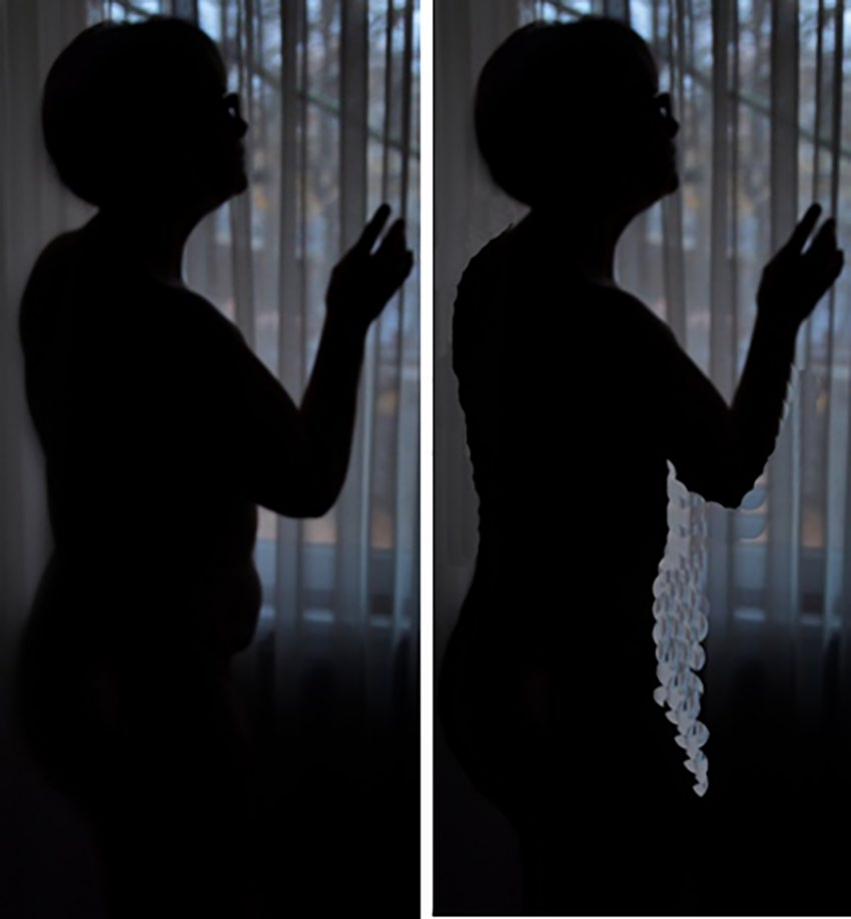
Photograph of before and after photoshopped image.

In this quotation, she describes altering the physical shape of her body (see Figure 7), demonstrating competing narratives between advocacy and understanding of media literacy and editing her own images.

Two women acknowledged the prevalence of beauty ideals depicted by media while also understanding the importance of not dwelling on unachievable appearance features. For instance, one said,

All the media, everything you see on TV and magazines and what not encourages us to remain looking youthful. And that bothers me a lot, because of the media altering the photos and everything and the same way it affects most women. But at the same time, I succumb to that to the degree that I continue to dye my hair. And I’m sure, my mother has beautiful white hair, and I’m probably about 85 percent gray.

**Figure 8.**
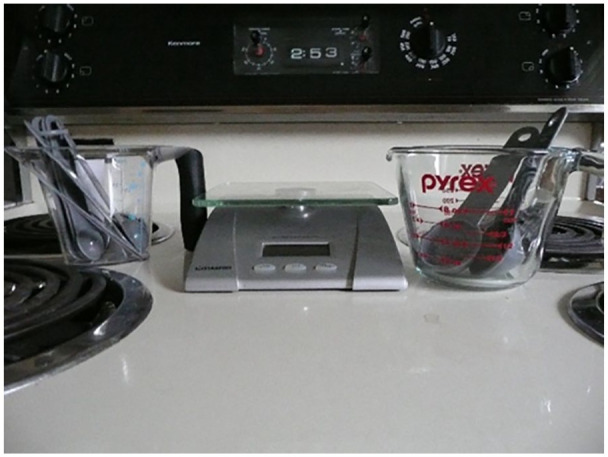
Photograph of scale and measuring cups used for new diet.

Another discussed an effort to appreciate her body and living with multiple sclerosis. However, for most of the interview she described her complicated relationship with her body weight. She took two photographs of a scale and measuring cups (e.g., Figure 8) she uses because her new diet entails measuring everything she eats. Although she strives to find acceptance and appreciation for her body as it is, she is regularly conflicted by the desire for a thinner body.

Another woman who had multiple sclerosis also found herself straddling a paradox between wishing and wanting to accept her body, while simultaneously buying into beauty discourses. She said,

Yeah, I should get over it, I really should. Because there’s so many bad things in the world and nobody really cares. And I think that the way things are getting, it’s easier to let go. And I listen to what other people are saying about me. Like you’re so nice, and kind, and that kind of stuff, that’s the stuff that really matters. And then I go back and I say, I wish I had nice shaped legs. But the other stuff, not too bad, but I really sort of wish I had my sister’s little tiny nose and my mother’s legs.

She slowly came to the realization that there are characteristics such as personality that should be more important than superficial appearance features you cannot control, yet she is unable to fully embrace that belief.

### Uncovering Positive Body Image and Resilience

**Figure 9.**
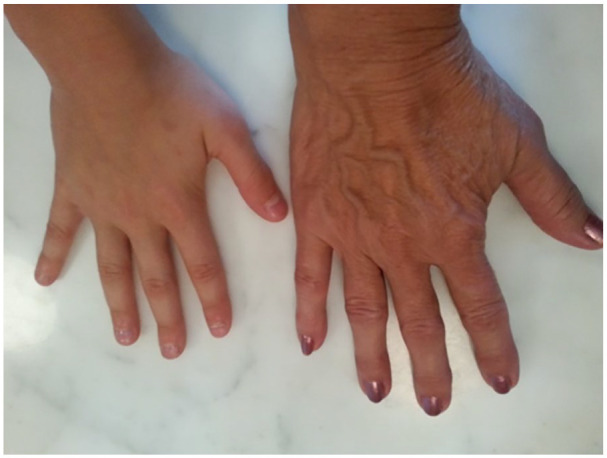
Photograph of grandmother’s and granddaughter’s hands.

Throughout the course of some interviews, participants came to realize their overly critical analysis of their appearance. Upon this reflection, some women recognized positive body image when describing their images. For example, one woman took a photo of her hand and her granddaughter’s hand and used this method as a teaching moment for her granddaughter (see Figure 9). Her granddaughter asked about all the veins in her grandmother’s hands. Her response to her granddaughter was that it is part of the natural aging process:

And my response is be realistic, I’m supposed to look this way, and my 93-year old mother reached a point where she never wanted to go sleeveless outside because of loose skin and I just repeatedly told her, mom your supposed to look like this . . . and this is what I try to convey to my granddaughter, she’s 11, I’m supposed to look like this.

One woman used a photograph of her on a leg press machine at Power Cord and said, “I love the definition in the legs! I have said attractive because I couldn’t come up with something but it just makes me feel powerful!” Although this image was chosen mainly for appearance reasons (i.e., the appearance of her leg), she used words such as “powerful,” demonstrating appreciation for her body.

Another woman exemplified resilience when she said, “. . . it’s all about what my body can do more than what my body looks like on the surface . . . I can feel afraid and still do it, still do things.” This quotation demonstrates a moment of appreciation for her body, rather than always criticizing her posture. Near the end of the interview, she came to the realization that “. . . when I really looked at the other photographs, I thought, you know, it’s probably not as bad as I’m thinking it looks, you know? I should lighten up on that.” This demonstrates the potential for photo-elicitation to lead to deep reflection and critical insights for moments of positive body image and resilience.

## Discussion

This study explored the body image experiences of middle-to-older age women with and without multiple sclerosis using the interview and photo-elicitation methods. Body image scholarship has evolved substantially over the years, with increased use of varying qualitative and quantitative methods. One method that is less used and understood in body image scholarship is photo-elicitation. Photo-elicitation has been argued to provide a promising opportunity for qualitative interviews to yield richer and more evocative findings (Collier, 1957), which is consistent with what we found in this study.

Overall, the women described complex and symbolic body image experiences, which often reflected their multiple identities and accompanying sociocultural structures across gender, age, and disability. These reflections by the women included overlapping stigmatized experiences (e.g., disability and weight stigma) such as one participant’s concern about people staring at and stigmatizing her in the scooter. We can contextualize this experience within the seminal work of Rosemarie Garland-Thomson (2009) who unpacks what it means to “stare” and be “stared at.” Although the fraught experience of being stared at results in some people trying to hide or erase their disability (e.g., clothing or medical intervention), one participant preferred emphasizing her disability to mitigate the stigma related to her body size. This finding demonstrates the potency of society-imposed weight stigma in relation to her disability and overall body image experiences. The significance of weight bias may reflect her experience of aging *with* disability (Aubrecht et al., 2020), where her relationship with disability may have been more familiar or comfortable; however, the stigma associated with her weight caused her great distress.

Another manifestation of “staring” could be the objectifying gaze. According to objectification theory (Fredrickson & Roberts, 1997), women (in particular) are socialized to experience routine sexual objectification and the objectifying gaze, which in turn teaches women to self-objectify and closely monitor their bodily appearance. Many women in this study evidenced self-objectification by, for example, photographing different parts of their body to evaluate and discuss. For instance, one woman captured photographs of arthritis in her hands and feet, illustrating her experience of aging *into* disability, and explained the inability to wear certain shoes anymore as she ages. However, her primary concerns during the interview were about the appearance of her arthritis as seen in her photographs, which she criticized quite bluntly.

Furthermore, many participants shared their body image concerns through the use of symbolism. This finding mirrors previous research (Nash, 2014) of pregnant women who thoughtfully curated images of objects that reflected how they felt about their bodies. This study extends previous literature, by demonstrating how the use of symbolism can capture the complex gender and aging intersection. For instance, one woman used the photograph of vibrant kayaks in fog to represent her daily negotiation with pressures of youthful beauty as she ages while wanting to accept her body and teach her granddaughter to do the same.

We found that some women scrutinized, sometimes quite harshly, their appearance—which was reflected in their choice of photographs and discussions. For example, women took pictures of specific body parts and compared them with contemporary beauty ideals or their younger selves. There is a plethora of research demonstrating the potential harmful impact that social comparison with idealized images can have on one’s body image (e.g., Calogero et al., 2007; Hargreaves & Tiggemann, 2004). On the contrary, some women became aware of their tendency to overly criticize their appearance, and during an opportunity of critical reflection during the interview, came to the realization they should be kinder to their bodies, regardless of appearance messaging in society. In Wood-Barcalow and colleagues’ (2010) holistic model of positive body image, they demonstrated that body image filtering (e.g., allowing body affirming information in and blocking or reframing body criticisms) may not be a “perfect” mechanism, but does serve to help protect against the inundation of harmful body-related information. The women in this study demonstrate this imperfect filtering of conflicting and paradoxical body image messaging.

### Photo-Elicitation as Method in Body Image Research

Although the primary interest in this study was to understand body image in aging women with and without multiple sclerosis, we cannot ignore the use of photo-elicitation in understanding the complexities of body image. Using the photo-elicitation method with interviews, we found that conversations with participants were richer and flowed more easily than in our previous body image research using interviews alone. Not only did participants have another medium to describe their body image experiences but they also had time to reflect deeply about their bodies during the process of collecting and sharing these images. For instance, one participant took the approach of staging items together, which took considerable creativity, and clearly prepared discussion points for the upcoming interview. It may have been easier for the women to visually represent these types of body image struggles than automatically put them into words (i.e., on the spot in an interview). Therefore, photo-elicitation might be an ideal strategy to use for researchers who are novice to interviewing because the photographs aided in body image discussions thereby relieving pressure from the interviewer and evoking rich discussion. A salient finding that was clear from using the photo method was the commonly experienced ingrained and normative views of the body, particularly about appearance. This may be partially explained by the very nature of photographs being a visual medium; regardless, every single participant, despite their age or relationship with disability, explained a complicated negotiation with the aesthetic dimension of their body image—demonstrating this method can tap into shared and differing experiences across a heterogeneous sample.

Another important implication from this study was the breadth of approaches taken by participants to capture their body image experiences using photographs. Many participants described difficulty in selecting photos that captured their body image well. Furthermore, some participants voiced concern about doing the study “incorrectly” or selecting the “wrong” images, thereby believing they were not contributing to the research. Also, while balancing creative freedom for participants, researchers may want to provide more specific instructions than our broad approach in this study (i.e., select 10 photographs that capture your body image), to lessen ambiguity.

### Implications for Improving Body Image

Photo-elicitation could be another tool to help participants recognize how they reflect about or treat their bodies. This may include revelations of unconscious pervasive negative thoughts toward appearance. This type of consciousness raising can be an effective approach for improving body image. Photo-elicitation may also provide opportunities to enhance aspects of positive body image such as body acceptance. Furthermore, photo-elicitation methods may provide unique opportunities to explore complex social identities. There has been a push for positive body image researchers to consider the many social identities that intersect in complicated ways with an individual’s body image (e.g., Tiggemann, 2015). In this study, the women were able to successfully capture this complexity (e.g., disability, aging, and gender) using photographs.

Even though this study was based on a small sample of seven women, the number of photos they provided yielded rich dialogue, and the findings suggest some potential for photo-elicitation being used within body image program/intervention research. Albeit some participants used the method to criticize their appearance, others also realized and reflected on this tendency. This revelation occurred for some participants during the interview process and provided quite a powerful understanding of how they treat their bodies. For example, one participant was not even aware of her propensity to scrutinize her appearance, particularly her hands. Writing exercises, where participants reflect on aspects of their body that they appreciate (e.g., body function), have demonstrated promising improvements in participants’ body image (e.g., improved body appreciation; Alleva, Martijn, et al., 2015). Of course, we caution the use of visual mediums in body image research (Bordo, 1993) and encourage future scholars considering this method in intervention designs to provide support to carefully guide participants through a self-compassionate reflective process. Just as recent literature has demonstrated focusing on body functionality does not inherently result in positive outcomes (e.g., Mulgrew & Tiggemann, 2018; Vinoski Thomas et al., 2019), based on this study, photo-elicitation also has its limits and should be used with care.

Because photographs of bodies have the potential to elicit harmful reactions (e.g., social comparison, objectification), symbolism may be a useful alternative approach. In this study, some participants used symbolism to unpack their body image experiences in the interview. Symbolism might be another useful strategy in body image interventions or programs to help facilitate delving into difficult conversations (e.g., appearance concerns or disability) creatively. Symbolism may act as a mechanism to initiate group discussion when the topic is sensitive in nature. Body image programs utilizing an in-person group format with a facilitator present (Alleva, Sheeran, et al., 2015) have shown to be the most effective at improving body image. However, in group formats, initiating group discussion and participant sharing of experiences, can be challenging; thus, researchers may consider symbolism as a conversation aid.

Finally, photo-elicitation could also be used as a psychoeducational tool. For example, one participant in this study talked about using her photograph of her hands and her granddaughter’s hands as a platform to teach about positive body image and aging. Psychoeducation is an important component of body image programs (e.g., Alleva, Sheeran, et al., 2015; Bailey et al., 2017, 2019), and photo-elicitation may be an effective medium to teach people across the lifespan the importance of media literacy, body appreciation, and other complex body image experiences such as stigmas associated with aging and disability.

## Conclusion

In conclusion, women in this study shared their dynamic, and even sometimes paradoxical, body image experiences across the aging–disability nexus while simultaneously contending with unrealistic youthful appearance standards. Significantly, this study highlighted the complexity of body image across multiple social identities (e.g., gender, age, weight status, disability) and how the intersection of these identities must be examined. Qualitative researchers should continue to explore the utility of photo-elicitation in other diversely embodied samples (e.g., comparison of the various types of multiple sclerosis and how they are impacted by various socially constructed roles, such as wife, mother, employee, woman, and more) and investigate other avenues of this approach, such as the difference in reflection processes based on the curation of newly captured versus older (e.g., album) photographs.
